# Benchmarking Nanopore Sequencing for CLN2 (TPP1) Mutation Detection: Integrating Rapid Genomics and Orthogonal Validation for Precision Diagnostics

**DOI:** 10.3390/ijms26115037

**Published:** 2025-05-23

**Authors:** Betül Teker, Gökce Akan, Hasan Hüseyin Kazan, Özge Özgen, Suzin Tatonyan, Mehmet Cihan Balci, Meryem Karaca, Fulya Kurekci, Edibe Pembegül Yıldız, Olcay Güngor, Adnan Deniz, Asuman Gedikbasi, Fatmahan Atalar, Gülden Fatma Gokcay, Mehves Poda

**Affiliations:** 1 Institute of Health Sciences, Istanbul University, 34452 Fatih, Türkiye; btltkr24@gmail.com (B.T.); suzintatonyan@gmail.com (S.T.); 2 Research Center of Experimental Health Sciences, Near East University, 99138 Mersin, Türkiye; gokce.akan@neu.edu.tr; 3 Department of Medical Biology, Gulhane Faculty of Medicine, University of Health Sciences, 06018 Ankara, Türkiye; hasanhuseyinkazan@gmail.com; 4 Rare Diseases Research Laboratory, Istanbul Medical Faculty, Istanbul University, 34452 Fatih, Türkiye; drozgozgen@gmail.com (Ö.Ö.); mehmetcbalci@hotmail.com (M.C.B.); meryemkrc@yahoo.com (M.K.); adnandeniz85@hotmail.com (A.D.); fatmahan.atalar@gmail.com (F.A.); guldengokcay@gmail.com (G.F.G.); 5 Division of Nutrition and Metabolism, Department of Pediatrics, Istanbul Medical Faculty, Istanbul University, 34452 Fatih, Türkiye; 6 Division of Neurology, Department of Pediatrics, Istanbul Medical Faculty, Istanbul University, 34452 Fatih, Türkiye; fulya.kurekci@istanbul.edu.tr (F.K.); edibe.pempegulyildiz@istanbul.edu.tr (E.P.Y.); 7 Neurology Unit, Department of Pediatrics, Pamukkale University, 20160 Pamukkale, Türkiye; olcaygungor@pau.edu.tr; 8 Neurology Unit, Department of Pediatrics, Medical School, Kocaeli University, 41001 Kocaeli, Türkiye; 9 Department of Pediatric Basic Sciences, Institute of Child Health, Istanbul University, 34452 Fatih, Türkiye; asuman.gedikbasi@istanbul.edu.tr; 10 Department of Rare Diseases, Institute of Child Health, Istanbul University, 34452 Fatih, Türkiye; 11 Department of Genetics, Aziz Sancar Institute of Experimental Medicine, Istanbul University, 34452 Fatih, Türkiye

**Keywords:** TPP1, CLN2, long-read sequencing, TPP1 enzyme activity, comprehensive care, DNA sequencing

## Abstract

CLN2 disease (neuronal ceroid lipofuscinosis type 2) is an ultra-rare lysosomal storage disorder caused by mutations in the TPP1/CLN2 gene, resulting in impaired tripeptidyl peptidase 1 (TPP1) activity. The timely initiation of enzyme replacement therapy is pivotal for attenuating progressive and irreversible neurodegeneration. This study aimed to benchmark the performance of Oxford Nanopore long-read sequencing (ONT-LRS) for targeted *TPP1* mutation detection in a Turkish CLN2 cohort and to assess its concordance with orthogonal validation methods, including Sanger sequencing and enzymatic activity assays. Using a custom-designed primer panel, the entire TPP1 gene (6846 bp) was sequenced on the Oxford Nanopore (ONT) MinIon platform in seven clinically confirmed CLN2 index patients and sixteen unaffected family members. Detected variants were validated via Sanger sequencing and correlated with TPP1 enzyme activity in leucocytes and dried blood spots. Four pathogenic or likely pathogenic *TPP1* variants were identified: c.622C>T (p.Arg208*), c.857A>G (p.Asn286Ser), c.1204G>T (p.Glu402*), and c.225A>G (p.Gln75=), along with fourteen additional benign variants. Variant allele frequencies were 50% for c.622C>T, 28.6% for c.1204G>T, 14.3% for c.857A>G, and 7.1% for c.225A>G. Notably, this is the first report to document the homozygous state of the c.857A>G variant and the compound heterozygous configuration of the c225A>G and c.622C>T variants in CLN2 patients, thereby expanding the known mutational landscape. In contrast, the globally common variant c.509-1G>C was not observed, suggesting regional variation in *TPP1* mutation patterns. Consistent with the prior Turkish studies, c.622C>T (p.Arg208*) was the most prevalent variant, followed by c.1204G>T (p.Glu402*). TPP1 enzymatic activity was significantly reduced in all affected individuals (*p* < 0.0001), supporting the functional relevance of the identified variants. ONT-LRS offers a robust, cost-effective platform for high-resolution analysis of the *TPP1* gene. Integrating molecular and biochemical data improves diagnostic precision and supports timely, targeted interventions for CLN2 disease, particularly in regions with high consanguinity and limited diagnostic infrastructure.

## 1. Introduction

Neuronal ceroid lipofuscinosis type 2 (CLN2) disease (OMIM 204500), also known as Jansky–Bielschowsky, represents an exceptionally rare lysosomal storage disorder within the neuronal ceroid lipofuscinosis (NCL) family, collectively termed Batten disease. Previously known as late-infantile neuronal ceroid lipofuscinosis (LINCL), CLN2 disease is an early childhood onset disorder caused by autosomal recessive mutations in the TPP1 gene (GenBank accession no. NM_000391.3) localized on chromosome 11p15. These mutations result in deficient activity of the lysosomal exopeptidase tripeptidyl peptidase 1 (TPP1) (EC 3.4.14.9) [1]. Similar to other NCL disorders (CLN1–CLN14), CLN2 disease involves lysosomal dysfunction, which culminates in the accumulation of autofluorescent storage materials, subsequent neuronal loss, and neurodegeneration. However, the precise in vivo substrates and the complete pathologic mechanisms remain incompletely characterized [2].

Pathogenic TPP1 gene variants, including splice site, missense, and nonsense mutations, as well as small deletions or insertions, primarily result in diminished or absent enzyme activity, impaired neuropeptide degradation, and the accumulation of subunit c of ATP synthase. This results in the lysosomal accumulation of ceroid lipofuscin, glial activation, and neuronal loss [3]. These mutations lead to reduced or absent TPP1 activity, resulting in lysosomal accumulation of ceroid lipofuscin, a hallmark of the CLN2 phenotype [4,5,6]. Ultrastructural analysis of lysosomal storage in CLN2 disease reveals a characteristic curvilinear profile [6,7]. Clinically, symptom onset correlates with peak TPP1 expression (ages 2–4) and includes new-onset seizures, ataxia, and a history of language delay [8]. Emerging research also suggests a potential link between TPP1 deficiency and oxidative stress [9].

To support clinical diagnostics and research, international databases, such as the International NCL Database and the University College London (UCL)-based NCL Mutation Database (http://ucl.ac.uk/ncl-disease/; accessed on 26 November 2024), catalog *TPP1* gene mutations across global cohorts [10]. Epidemiological data, primarily from Western countries, report an incidence of 1–3 per 100,000 and a prevalence of 2–4 cases per 1,000,000 [11,12,13,14,15,16,17]. Updated guidelines in 2021 refined diagnostic criteria, clinical assessments, and management practices for CLN2 disease [18,19].

Despite these advances, the limited availability of enzyme assays and molecular diagnostic tools significantly hampers early and accurate diagnosis, particularly in regions with high rates of consanguinity, where ultra-rare disorders like CLN2 are prevalent. However, the lack of widespread access to molecular diagnostic tools and enzyme assays remains a critical barrier to early and accurate diagnosis. Definitive diagnosis requires the identification of pathogenic TPP1 variants and the confirmation of deficient TPP1 enzymatic activity (e.g., in leukocytes, fibroblasts, or dried blood spots) [20]. In resource-constrained settings, either biochemical or molecular confirmation alone is diagnostically valuable [20]. Historically limited to symptomatic management, therapeutic prospects dramatically improved with the approval of enzyme replacement therapy cerliponase alfa in 2017 (Brineura^®^, BioMarin Pharmaceutical Inc., Novato, CA, USA), significantly decelerating disease progression [21,22]. Nevertheless, early diagnosis remains critical for optimal treatment outcomes, emphasizing the necessity for enhanced epidemiological studies and improved diagnostic capabilities to advance patient care and quality of life.

Conventional sequencing approaches, such as Sanger sequencing or short-read next-generation sequencing (NGS), often fail to detect intronic, structural, or phasing-relevant variants that may affect diagnostic sensitivity in rare monogenic disorders. Oxford Nanopore long-read sequencing (ONT-LRS) offers several advantages, including long-read capacity, real-time data output, and lower infrastructure requirements, making it especially suitable for use in decentralized or resource-limited settings.

In this study, ONT-LRS was employed using a custom amplicon panel spanning the entire *TPP1* gene to investigate the mutational landscape in a Turkish CLN2 cohort. Its performance was benchmarked against orthogonal validation methods, namely Sanger sequencing and TPP1 enzymatic activity assays, to assess both analytical accuracy and functional relevance. This integrative approach aligns with precision diagnostic principles by ensuring robust molecular and biochemical confirmation of pathogenicity. By combining high-resolution variant detection with enzyme activity profiling, this study aims to enhance diagnostic accuracy and support timely therapeutic interventions in this devastating pediatric neurodegenerative disorder.

## 2. Results

### 2.1. Study Population and TPP1 Enzyme Activity

This study involved patients with clinical presentations suggestive of CLN2 (n = 7) who had confirmed diagnoses via genetic or enzymatic testing and were undergoing enzyme replacement therapy (ERT). Additionally, 16 first-degree relatives were included for comparative analysis.

**Figure 1:**
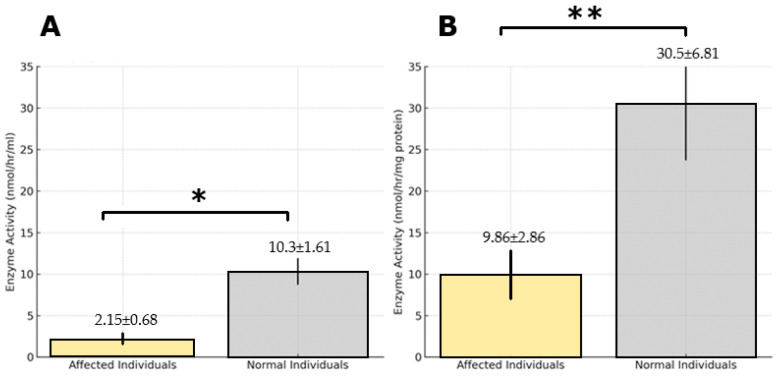
TPP1 activity in CLN2 patients and healthy controls. This figure illustrates the study population and TPP1 enzyme activity levels in patients with clinical presentations suggestive of CLN2 and their first-degree relatives. This study included seven index patients diagnosed with CLN2 based on genetic or enzymatic testing, along with 16 first-degree relatives. (**A**) TPP1 enzyme activity analysis of dried blood spot (DBS) samples. (**B**) TPP1 enzyme activity in leukocyte samples. Data are shown as means and SDs (affected individuals n = 7, normal individuals n = 16). * *p* < 1 × 10^−8^, ** *p* < 1 × 10^−4^. Abbreviations: DBS: dried blood spot; TPP1: tripeptidyl peptidase 1; CLN2: ceroid lipofuscinosis, neuronal 2.

Enzyme activity measurements from dried blood spot (DBS) samples revealed significantly reduced TPP1 activity in affected individuals, with a mean activity of 2.15 ± 0.68 nmol/h/mL (range: 0.8–3.5), compared to 10.3 ± 1.61 nmol/h/mL (range: 7.1–13.5) in healthy subjects. The TPP1 activity in affected individuals was strongly diminished as compared to healthy subjects (*p* < 1 × 10^−8^). Similarly, in leukocyte samples, the mean activity of the TPP1 enzyme was (9.86 ± 2.86 nmol/h/mg protein) markedly diminished in comparison to normal subjects (30.5 ± 6.81 nmol/h/mg protein). The difference between healthy subjects and patients was also statistically significant (*p* < 1 × 10^−4^). These results from DBS and leukocyte samples strongly corroborated the molecular genetic findings, confirming the effect of the detected mutations in the TPP1 activity of affected individuals, as illustrated in Figure 1A,B.

The precision of the enzyme activity assay was assessed through intra- and interassay coefficients of variability (%CV). For the leukocyte samples, the intra-assay %CV was 4.32%, and the interassay %CV was 7.13%. For the DBS samples, the intra- and interassay %CV values were 6.05% and 7.01%, respectively. Reference ranges for TPP1 enzyme activity, established from the venous and capillary blood samples of age- and sex-matched healthy volunteers, were 27.3 ± 5.7 nmol/h/mL for leukocytes and 29.3 ± 4.02 nmol/h/mL for DBS, serving as critical benchmarks for distinguishing normal from deficient enzyme activity.

### 2.2. Mutations in the TPP1 Gene Identified via ONT-LR Sequencing and Sanger Sequencing Validation

**Figure 2:**
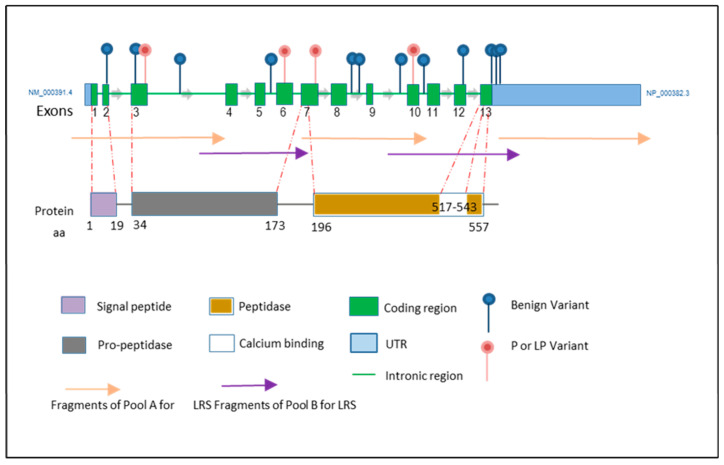
Overview of the CLN2 gene and detected variations. This figure presents a schematic representation of the TPP1 gene (NCBI RefSeq Annotation GCF_000001405.40-RS_2024_08), highlighting its exon–intron structure and key protein domains. It also illustrates the fragments generated from ONT-LRS. It includes the specific genomic regions targeted for ONT-LRS and captures the identified variations along the gene. The primer designs and corresponding fragments were optimized to comprehensively cover all relevant coding and regulatory regions of the TPP1 gene, ensuring the precise and accurate detection of pathogenic variants.

Following enzyme activity analyses, within the aim of this study, mutation profiling of the TPP1 gene was performed using the ONT-LRS platform, with subsequent validation through Sanger sequencing. The ONT-LRS workflow included base-calling, an analysis of Fastq files using Massive Analyzer v4.5.1 software, and the evaluation of candidate variants against public databases, including ClinVar [23], Franklin by Genoox (https://franklin.genoox.com, accessed on 26 November 2024), and VarSome [24]. This approach identified a total of three pathogenic (c.622C>T, c.857A>G, and c.1204G>T) and one likely pathogenic variant (c.225A>G) among seven index patients and their sixteen healthy family members. The locations of pathogenic and likely pathogenic variants, along with their relationships with the active regions of the protein, are presented in Figure 2. In the public databases, three variants were classified as pathogenic (P): c.622C>T (p.Arg208Ter), c.857A>G (p.Asn286Ser), and c.1204G>T (p.Glu402Ter), and one variant was classified as likely pathogenic (LP): c.225A>G (p.Tyr76Lysfs*10) (Figure 2, Table 1).

Among the six index patients, homozygosity was observed for three pathogenic variants: c.857A>G (one patient), c.1204G>T (two patients), and c.622C>T (three patients) (Figure 2). One patient exhibited compound heterozygosity with the c.622C>T and c.225A>G variants. Notably, the c.1204G>T variant did not have a corresponding SNP (rs) code in public databases and was classified as likely pathogenic. Consequently, two patients were homozygous for this variant. Both the c.1204G>T and c.622C>T mutations introduce a premature stop codon. All identified pathogenic and likely pathogenic variants were also confirmed through Sanger sequencing.

**Figure 3:**
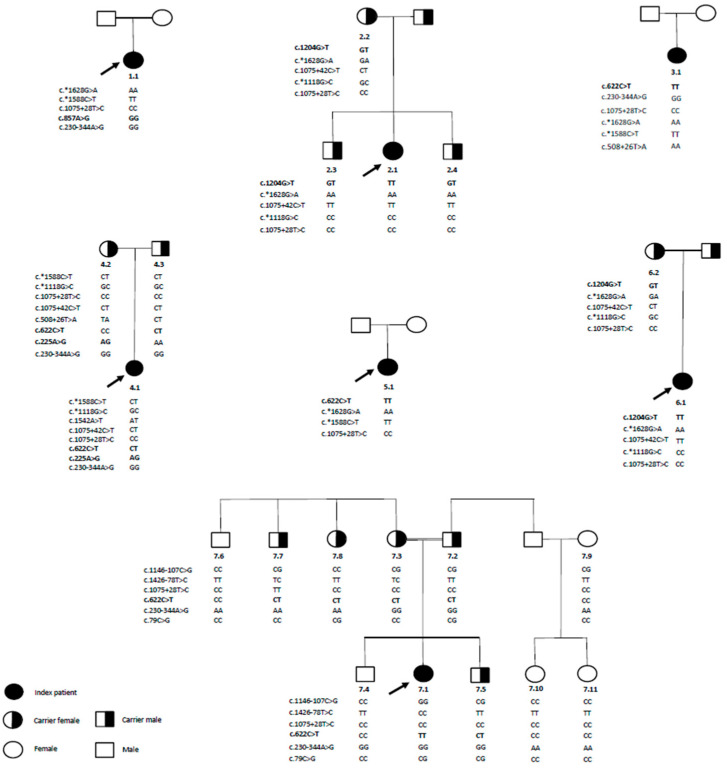
Pedigrees and phased genotypes derived from ONT-LRS. This figure depicts the pedigree charts and corresponding phased genotypes of families affected by CLN2 disease, as determined by ONT-LRS. The pedigrees illustrate the inheritance patterns and the specific genetic variants identified. The pathogenic (P) and likely pathogenic (LP) variants, as well as their segregation within the families, are shown to provide insight into the genetic architecture of CLN2 disease. The variants classified as pathogenic and likely pathogenic are bolded. Wild-type and mutant alleles are clearly represented, with bolded genotypes highlighting the inheritance of the mutant alleles. Index patients are indicated by arrows, representing the probands in each family.Symbols: Square: Male; Circle: Female; Filled Symbol: Affected individual; Half-filled Symbol: Carrier; Empty Symbol: Unaffected individual. Abbreviations: CLN2: Ceroid lipofuscinosis, neuronal 2; P: pathogenic; LP: likely pathogenic.

Supplementary Table S1 provides a detailed summary of the genotypes and respective variant locations of the TPP1 gene. Table 1 provides a detailed summary of the pathogenic variants identified in the TPP1 gene among seven CLN2-diagnosed patients. The figure highlights the genetic- and protein-level consequences of these variants, their allele frequencies as reported in public databases (gnomAD and TOPMed) [25,26], and their classification based on pathogenicity (pathogenic or likely pathogenic). Each variant’s mutation type, frequency, classification, allele count, and reference SNP (rs) number, when available, are also included. In addition to the pathogenic variants, ONT-LRS analysis identified 14 genetic alterations within the TPP1 gene, including one missense mutation, one frameshift mutation, six intronic mutations, one synonymous mutation, and three 3′UTR variants, all of which were categorized as benign (Supplementary Table S1). Genotypic and parental segregation analysis of the seven families is detailed in Figure 3, while the Integrative Genomics viewer (IGV) visualization of the pathogenic variants is depicted in Supplementary Figure S1. The c.857A>G variant is in a homozygous state in individual 1.1 (index patient) (Panel A). The c.225A>G variant (Panel B) was detected in family 4. The variant was detected in the index patient (4.1) and the mother (4.2) in heterozygous states, while the father (4.3) was a non-carrier. The c.1204G>T variant was detected in families 2 and 6 (Panel C). In family 2, the affected individual 2.1 is homozygous for the variant, while the mother (2.2) and siblings (2.3 and 2.4) are heterozygous. In family 6, the index patient (6.1) is homozygous, and the mother (6.2) is heterozygous. The c.622C>T variant was detected in families 3, 5, and 7 (Panel D). Index patients 3.1, 5.1, and 7.1 are homozygous for the variant. In family 4, the index patient (4.1) and the father (4.3) are heterozygous. In family 7, carriers include the father (7.2), mother (7.3), a brother (7.5), the maternal aunt (7.7), and the maternal uncle (7.8).

Overall, this study successfully identified four distinct pathogenic alterations in TPP1 among patients with CLN2 disease via the ONT-LRS. A total of 14 variants within the TPP1 gene were identified, including the three classified as pathogenic and one as likely pathogenic. The ONT-LRS platform demonstrated complete concordance (100%) with conventional diagnostic methods, with all pathogenic variants validated by Sanger sequencing, confirming the accuracy and reliability of Nanopore sequencing for molecular characterization. Furthermore, significant reductions in TPP1 enzyme activity in affected individuals compared with healthy controls supported the molecular findings, highlighting the robust utility of this integrated diagnostic approach for CLN2 disease.

## 3. Discussion

This study highlights the diagnostic utility of ONT-LRS in identifying and characterizing both known and novel *TPP1* variants in the Turkish CLN2 cohort. Using a streamlined, long-read sequencing workflow, four pathogenic or likely pathogenic variants (c.622C>T, c.857A>G, c.1204G>T, and c.225A>G), along with 14 additional benign variants, were identified across seven index cases and 16 unaffected relatives. The integration of ONT-LRS with TPP1 enzyme activity assays confirmed the pathogenicity of these variants, reinforcing the diagnostic power of combining molecular and biochemical approaches.

The ONT-LRS data revealed the identification of these variants in various genetic contexts: c.857A>G was identified in one case in the homozygous state, c.1204G>T in two cases, and c.622C>T in three cases. Notably, one patient exhibited compound heterozygosity for the c.622C>T and c.225A>G variants.

The c.622C>T (p.Arg208Ter) and c.1204G>T (p.Glu402Ter) variants, known for introducing premature stop codons, were particularly prevalent, aligning with previous reports that cite c.622C>T as a common mutation in CLN2 disease [27,28,29,30,31]. It also affects RNA splicing and is classified as pathogenic in the ClinVar database. In this study, this variant was identified in a homozygous state in the index cases of families three, six, and seven. Furthermore, in the index case of family four, this variant was identified in compound heterozygosity with another variant.

The c.857A>G (p.Asn286Ser, rs119455958) variant, another pathogenic mutation reported in ClinVar, was identified in a homozygous state in the index case of family one. This mutation results in the substitution of asparagine with serine at codon 286, which is located in a key N-glycosylation site of the TPP1 protein [32]. This missense variant has been shown to impair TPP1 function in individuals with neuronal ceroid lipofuscinosis [33]. A comprehensive analysis of four missense mutations (p.Asn286Ser, p.Ile287Asn, p.Thr353Pro, and p.Gln422His) revealed that all mutations cause localization defects, hindering the maturation of the peptidase [31,33].

c.225A>G (p.Gln75=, rs368709098), a rare variant previously reported in only two cases [33], was identified in heterozygous state in the index case from family four, together with c.622C>T as the other pathogenic allele. According to SpliceAI [34], a deep learning-based tool that predicts splice site disruption, this variant has a high score of 0.73 (range 0–1, with scores closer to 1 indicating stronger splice-altering potential). Located in exon 3 of the *TPP1* gene and positioned only four nucleotides from the adjacent intron, this variant is predicted to create an alternative splice site, resulting in a frameshift and a premature stop codon (p.Tyr76Lysfs*10). The c.1204G>T (p.Glu402Ter) variant, the rarest reported, was found to be homozygous in index cases from families two and six. It introduces an early stop codon at position 402, meeting the ACMG/AMP criterion PVS1 (pathogenic very strong) according to the Franklin database. Although absent from ClinVar, it is classified as pathogenic in the UCL database [33] and was previously linked to CLN2 disease by Kousi et al. [35]. Familial segregation analysis confirmed the heterozygous carrier status in the index patient’s mother and healthy siblings.

These findings align with a recent Turkish study involving 30 patients, which reported homozygous mutations in 88% of cases, with c.622C>T (p.Arg208*) being the most common variant detected in 40% of families and c.1204G>T (p.Glu402*) in 15% [36]. Another Turkish study by Köse et al. (2021) further identified c.686A>T (p.Glu229Val) as a pathogenic variant [37]. Additionally, the c.857A>G variant was identified in the homozygous state (reported in the heterozygous state in the UCL-Database), marking the first report of this mutation in Turkish patients with CLN2 disease. These results underscore the importance of regional genetic studies in elucidating population-specific mutation profiles.

In a comprehensive review of TPP1 gene variants, Gardner et al. [11] evaluated data of 460 individuals worldwide by combining results from the UCL TPP1 locus-specific database with literature searches. Their analysis identified 155 unique variants for TPP1, with c.509–1G>C (27%) and c.622C>T [p.(Arg208*)] (23%) being the most frequently reported globally, whereas the allelic frequency of p.Asp276Val was only 2%. Thus, compared with our results, c.622C>T (p.Arg208*) appears to be a region-specific variant of relevance for CLN2 diagnosis in Türkiye. Notably, until 2023, neither c.509-1G>C nor c.622C>T (p.Arg208*) had been identified in individuals from Southeast Asian or Middle Eastern populations [38], suggesting that these mutations are not universally prevalent. Moreover, distinct mutation profiles have also been reported in specific regions, such as Argentina, South America [39,40] and Newfoundland, Canada [41,42], where founder effect mutations are well characterized. The 2023 study from Iran, which focused on NCLs, detected 18 mutations across several NCL-related genes among 29 patients using whole-exome sequencing [38]. For CLN2 disease specifically, c.622C>T was identified in two patients, while other mutations included c.509-1G>C, c.887G>A, c.1243del, c.1106C>T, and c.509-2A>G. Their study confirms that c.622C>T is the most frequently detected mutation in the region [36,37,38].

In this study, ONT-LRS was employed as a novel technology to achieve comprehensive coverage of the entire TPP1 gene. When two pathogenic mutations are identified, it is crucial to confirm that they are located on separate parental alleles (i.e., in trans), which is achieved through parental segregation analysis. This approach also helps detect potential allele dropout, preventing false assessments of homozygosity [43]. While de novo germline mutations are theoretically possible, none have been reported to date, and large deletions remain rare [29]. In the case where only one pathogenic allele is identified, enzyme activity testing becomes essential, as the absence of a second mutation does not exclude CLN2 disease. Additionally, molecular analysis may reveal variants of uncertain significance (VUSs). Among the 14 sequence alterations detected in this study, 10 (71.4%) were classified as likely nonpathogenic, with only 1 VUS identified. As molecular testing technologies advance, the number of documented VUSs may increase. To ensure accurate diagnoses, pathogenic variants identified through molecular analysis should be evaluated with enzyme assay testing to confirm their functional role in the disease [44].

Further highlighting the clinical utility of the current approach, TPP1 enzyme activity assays performed on leukocytes, fibroblasts, or dried blood spots (DSBs) validated the pathogenic impact of the identified mutations, further strengthening the “pathogenic” classification of the detected variants and emphasizing the value of enzyme testing for mutation-specific characterization. By recognizing this variant as pathogenic, healthcare providers can offer more precise genetic counseling and implement targeted interventions for both patients and families.

This study’s findings highlight that integrating molecular and biochemical analyses offers a comprehensive strategy for assessing both established and novel TPP1 mutations. This approach is particularly valuable in cases involving homozygous, heterozygous, as well as likely pathogenic (LP) variants and variants of uncertain significance (VUSs). By correlating these variants with enzymatic functionality, clinical outcomes can be predicted more accurately, thus enhancing diagnostic precision and facilitating personalized treatment strategies.

As a diagnostic tool for CLN2, enzyme activity assays may be useful mainly in cases in which the physician already has a strong suspicion of the disease. Some limitations should also be considered; DBSs offer a practical and easily transportable option for enzymatic analysis, although deficient TPP1 activity in DBSs requires confirmation through molecular analysis for definitive CLN2 diagnosis. While TPP1 activity measurement in leukocytes or fibroblasts serves as a confirmatory test, it necessitates specialized laboratory infrastructure for sample collection and preparation. Transporting these samples poses logistical challenges, especially over long distances, owing to specific handling requirements and regulatory constraints, a common issue in large countries such as Türkiye.

In summary, the current study underscores the diagnostic advantages of ONT-LRS, particularly in detecting novel and region-specific variants in the TPP1 gene associated with CLN2 disease. The integration of ONT-LRS with biochemical assays represents a comprehensive approach that enhances the understanding of the mutation spectrum and improves diagnostic accuracy. These findings provide valuable insights into CLN2 pathogenesis and offer a pathway for more precise and tailored management for affected individuals and their families.

## 4. Materials and Methods

### 4.1. Study Design and Sample Collection

This study focused on patients with clinical presentations suggestive of neuronal ceroid lipofuscinosis type 2 (CLN2) who had confirmed diagnoses through genetic and enzymatic testing and were undergoing enzyme replacement therapy (ERT).

#### 4.1.1. Sample Collection

Dried blood spot (DBS) and EDTA whole blood samples were obtained from the study participants referred to the Division of Pediatric Nutrition and Metabolism at Istanbul Medical Faculty, University of Istanbul. The study cohort comprised seven pediatric CLN2 patients. To establish normal reference values, control samples were collected from healthy children.

#### 4.1.2. DBS Sample Preparation

DBS samples were collected via standard procedures; blood was applied to a Whatman 903 card, which was air-dried for at least 4 h and then stored in individual plastic bags under appropriate conditions. Additionally, 4 mL of EDTA whole blood was collected from each patient for downstream analyses.

#### 4.1.3. Ethical Compliance and Data Collection

Ethical approval was granted by the Istanbul Medical Faculty Ethics Committee, and written informed consent was obtained from all participants or their legal guardians. Demographic and relevant clinical information was collected from patients (n = 7) diagnosed with CLN2 and first-degree relatives (n = 16) before obtaining blood and serum samples for further analysis.

### 4.2. Biochemical Measurements

The protein assay dye reagent concentrate was procured from Bio-Rad, while all other chemicals and substrates used in the study were sourced from Sigma-Aldrich (St. Louis, MO, USA). Specific enzyme activities for TPP1 (CLN2) in both DBSs and leukocytes were measured via fluorometric methods (Synergy HTX Multimode Reader-Biotek, Agilent, CA, USA). To ensure sample quality, a reference enzyme, β-galactosidase activity, was also measured in all the assays. The samples were processed within one week of collection. DBS samples were tested within 72 h and stored at −20 °C, whereas total leukocytes were isolated from EDTA whole blood samples on the delivery day and stored at −20 °C until further use. Total leukocytes were extracted with red cell lysis buffer (RCLB) containing 0.15 M ammonium chloride, 0.01 M potassium bicarbonate, and 0.1 mM disodium EDTA at pH 7.2. The leukocytes were suspended in 0.9% sodium chloride and sonicated in an ultrasonic sonicator processor (TF-250 N, Tefic Biotech, Longfubeijun, China) for 10 s. The Bradford method was utilized to quantify the proteins in leukocytes. The microassay procedure for microtiter plates was performed according to the manufacturer’s instructions (Bio-Rad #5000006). TPP1 (EC 3.4.14.9) and reference enzyme β-galactosidase (EC 3.2.1.23) activity measurements in DBSs and leukocytes were carried out via the methods described by Rodrigues et al. [45] and Civallero et al. [45]. TPP1 activity [46] was measured in DBSs and leukocytes as follows: For DBSs, a 3.2 mm disk was punched into a 96-well microplate and incubated for 20 h at 37 °C with 40 μL of the Ala-Ala-Phe-7-amido-4-methylcoumarin (AAF-AMC) substrate (0.5 mM) in acetate buffer (pH 4.0), along with EDTA, pepstatin A, and E64. The reaction was terminated with 200 μL of 0.13 M ethylenediamine (pH 11.3). For leukocytes, 15 µg of protein was incubated for 2 h at 37 °C with 20 μL of the AAF-AMC substrate and 40 μL of 0.425% sodium chloride, and the reaction was stopped with 300 μL of ethylenediamine. β-Galactosidase activity was used as a control; for DBS, the 4-MU-β-D-galactopyranoside (MUG) substrate (0.8 mM) was used, with the reaction performed in a similar microplate setup and stopped with 300 μL of ethylenediamine. For leukocytes, 15 µg of protein was incubated for 30 min at 37 °C with 50 μL of the MUG substrate (1 mM) and 0.584% sodium chloride, and the reaction was terminated with 0.13 M ethylenediamine. The fluorescence was measured via a Synergy HTX Multimode Reader, with emission at 460 nm and excitation at 355 nm. Measurements were corrected for blanks and compared to calibration curves for β-galactosidase (4-MU) and TPP1 (4-MC). Enzyme activities are expressed as nanomoles per hour per milliliter of blood (DBS) or nanomoles per hour per milligram of protein (leukocytes).

#### Protocols for Precision and Linearity Determination

To validate the method’s performance in analyzing CLN2, precision and linearity were determined according to the Clinical and Laboratory Standards Institute (CLSI) guidelines [47].

Precision was evaluated by calculating interassay and intra-assay variability coefficients (%CV). For this purpose, analyses were performed on both DBS and leukocyte samples over three consecutive days, as well as within the same day on three separate occasions, to account for day-to-day and within-day variability. Reference ranges for TPP1 enzyme activity were derived from venous and capillary blood samples obtained from a cohort of age- and sex-matched healthy volunteers (n = 10).

The enzyme activity in leukocyte samples was expressed in nanomoles per hour per milligram of protein (nmol/h/mg protein), whereas DBS sample activity was expressed in nanomoles per hour per milliliter of blood (nmol/h/mL). This approach ensured a comprehensive evaluation of the method’s precision and linearity, thereby providing robust validation of the accuracy and reliability of the CLN2 analysis protocol.

### 4.3. Genomic Analysis

#### 4.3.1. DNA Extraction

Five to ten milliliters of whole blood were collected from each patient in EDTA-containing blood collection tubes. DNA extraction was performed using the MobiomX DNA Isolation Mini Kit (Massive Bioinformatics, Istanbul, Türkiye) following the manufacturer’s protocol. All dsDNA samples were quantified using a Qubit™ 4 fluorometer (Thermo Fisher Scientific, Madison, WI, USA).

#### 4.3.2. Primer Design and Target Amplification

In silico primer design for long PCR amplification of the TPP1 gene (NM_ 000391) was conducted using the NCBI-Primer-BLAST tool [48]. To verify the reliability of the in silico predictions, the primers were tested experimentally against the human reference DNA. Long-term PCR was performed according to established protocols with the designed primer pairs. The resulting PCR products were subsequently analyzed via gel electrophoresis on a 2% agarose gel, allowing for the visualization and assessment of the amplicon sizes. The designed primer pairs and their locations within the genome are given in Table 2.

The primer pairs are designed for amplifying regions of the TPP1 gene and are tailored for Oxford Nanopore long-read sequencing (ONT-LRS). Each primer pair includes forward (F) and reverse (R) primers with melting temperatures (Tm), genomic binding coordinates, and the length of the amplified fragments. Table 1 also specifies the exons covered by each amplicon and their assignment to sequencing pools (A or B), enabling parallel sequencing and ensuring comprehensive variant detection with high accuracy. Genomic locations refer to the positions on the human genome assembly (GRCh38/hg38). The sequencing pools represent distinct sets of amplicons grouped for parallel sequencing analysis.

#### 4.3.3. Library Construction and Sequencing

Library construction and sequencing were conducted according to the methodologies outlined by Karamitros and Magiorkinis [49]. PCR products were utilized to generate a sequencing library using the Ligation Sequencing Kit (catalog number SQK-LSK109; Oxford Nanopore Technologies, London, UK) in accordance with the manufacturer’s guidelines. The PCR products underwent end repair and then tailing with the NEBNext End Repair/dA-tailing module. Barcode ligation was achieved using the NEBNext Ultra II Ligation Module Kit and PCR barcoding EXP-NBD196. Adapter ligation was subsequently performed with the NEBNext Quick Ligation Module Kit. The prepared library was then loaded onto an R9.4 flow cell (FLO-MIN106) and sequenced with the MinION Mk1C instrument via MinKNOW software version 22.10.7 (Oxford Nanopore Technologies) [50].

#### 4.3.4. Data Analysis

Statistical analysis of the enzyme assay data was performed via SPSS software (Statistical Package for the Social Sciences 25.0; SPSS Inc., Chicago, IL, USA). The quantitative data are expressed as the means ± standard deviations (S.Ds.), whereas the qualitative data are presented as percentages.

Basecalling and demultiplexing were carried out with Guppy software version 6.3.9 (https://pypi.org/project/ont-pyguppy-client-lib/6.3.9, accessed on 13 April 2025) with default settings [50]. The resulting Fastq files were further processed via Massive Analyzer (version 4.5.1, Massive Bioinformatics, Istanbul, Türkiye). Read quality was evaluated via FastQC (version 0.12.0) (http://www.bioinformatics.babraham.ac.uk/projects/fastqc/, accessed on 13 April 2025) [50]. The alignment was performed with MiniMap2 v2.24 [47] via a custom BED file. Assembly corrections were carried out via Medaka v1.12.0 (https://github.com/nanoporetech/medaka, accessed on 13 April 2025), and variant calling was performed via Clair3-Trio v0.7.1 [51]. The resulting VCF files were annotated via ANNOVAR (v3.1.2) [52]. Variant filtering was performed with VarAFT software v2.17-2 [53] by setting variant frequencies to less than 0.01%. Candidate variants were assessed against the ClinVar [23], Franklin by Genoox (https://franklin.genoox.com), and VarSome [24] databases. Unreported variants were classified according to the American College of Medical Genetics (ACMG) criteria [54]. Variants were prioritized based on population frequencies available in gnomAD [25] and TOPMed [26], focusing on those with minor allele frequencies of less than 0.1%. Allele frequencies were interpreted [55], with 20–70% indicating heterozygosity and greater than 70% indicating homozygosity.

## 5. Conclusions

This study highlights the transformative potential of ONT-LRS in achieving precise molecular diagnostics in CLN2 disease. A total of sixteen TPP1 variants were identified, including three pathogenic (c.622C>T, c.857A>G, and c.1204G>T) and one likely pathogenic variant (c.225A>G). Notably, the findings include novel and region-specific variants, with c857A>G being reported for the first time in homozygosity among our cohort. Although c.509-1G>C is frequently reported as a common variant globally, it was absent in our cohort, pointing to regional variations in CLN2 mutation patterns. Consistent with the findings from two other Turkish studies, c.622C>T (p.Arg208*) was the most prevalent variant in our cohort, followed by c.1204G>T (p.Glu402*). The molecular findings showed strong concordance with enzyme activity measurements, validating the robustness of the current integrated diagnostic approach. With minimal cost and rapid turnaround times, ONT-LRS not only confirmed known pathogenic variants but also revealed additional clinically significant mutations, offering profound insights into the genetic architecture of CLN2. This synergistic approach has the potential to enhance diagnostic precision, expand the ability of understanding of the disease, and ultimately improve patient outcomes.

## Figures and Tables

**Table 1:** Pathogenic variants identified in the *TPP1* gene among patients.

HGVSc	HGVSp	Frequency (gnomAD/TOPMed)	Classification	Type	# Affected Alleles	RS ID
c.622C>T	p.Arg208Ter	0.00022/0.00023	P	Stop codon	7	rs119455955
c.1204G>T	p.Glu402Ter	0.00000	P	Stop codon	4	-
c.857A>G	p.Asn286Ser	0.00000/0.00001	P	Missense codon	2	rs119455958
c.225A>G	p.Gln75 =	0.0002/0.00006	LP	Aberrant splicing	1	rs368709098

HGSVc: coding DNA sequence annotation based on Human Genome Variation Society (HGSV) guidelines; HGVSp: protein-level notation according to HGSV, indicating the predicted impact on the protein sequence; gnomAD: The Genome Aggregation Database; TOPMed: Trans-Omics for Precision Medicine. RS ID: Reference SNP (single nucleotide polymorphism) identifier assigned by the dbSNP database. P stands for “Pathogenic”; LP stands for “Likely Pathogenic”, #: number of detected alleles.

**Table 2:** Primer design for the amplification of TPP1 gene regions for ONT-LRS.

	Primer Pair	Sequence	Tm. (°C)	Genomic Location Start-End	Fragment Length (bp)	Exons	Seq. Pools
**1**	TPP1_6089_F	GGCCAGTAAGTTGCAAATGTCGCACC	67.1	11_6617790-6618015	1551	1-2-3	A
	TPP1_7639_R	CCACCCTTGCCTAGCATTTGGGACC	67.6	11_6619516-6619540			
**2**	TPP1_4859_F	GTCCAACCACACGGGCTACTGATGC	67.7	11_6616760-6616784	1512	4-5-6-7	B
	TPP1_6370_R	TTCACAGCAGGGGGAGTGTGTGC	67.2	11_6618249-6618271			
**3**	TPP1_3391_F	TGGGGGCTAGAGCTCAGGAACTTCG	67.6	11_6615292-6615316	1600	7-8-9-10-11	A
	TPP1_4990_R	ACCCACGATCTCTGCTCTGACTCCC	67.3	11_6616867-6616891			
**4**	TPP1_2167_F	GGAGAGGGAGTGGGCAACTATGATGG	66.2	11_6614068-6614093	1579	10-11-12-13	B
	TPP1_3745_R	ACCTGGGCTATACTCACCCCTCCC	66.8	11_6615623-6615646			
**5**	TPP1_793_F	GCTGTAGGAGGAGGAGGAGTTTCAGC	66.1	11_6612694-6612719	1502	13	A
	TPP1_2294_R	CTGCAAGGAGACCTCTACTGTCACCG	66.3	11_6614170-6614195			

## Data Availability

Further information related to the current study is available from the corresponding author upon reasonable request.

## Supplementary Materials

The following supporting information can be downloaded at https://www.mdpi.com/article/10.3390/ijms26115037/s1.

## Abbreviations

The following abbreviations are used in this manuscript:

AAF-AMC	Ala-Ala-Phe-7-amido-4-methylcoumarin
ACMG	American College of Medical Genetics
CLN2	Ceroid Lipofuscinosis, Neuronal 2
DBS	Dried Blood Spot
LP	Likely Pathogenic
NCL	Neuronal Ceroid Lipofuscinosis
ONT-LRS	Oxford Nanopore Technologies Long-Read Sequencing
TPP1	Tripeptidyl Peptidase 1
UCL	University College London
VUS	Variant of Unknown Significance
