# Supplementary material for: Benchmarking Nanopore Sequencing for CLN2 (TPP1) Mutation Detection: Integrating Rapid Genomics and Orthogonal Validation for Precision Diagnostics
Source: Int J Mol Sci. 2025 May 23;26(11):5037. doi: 10.3390/ijms26115037 (PMC12155472; doi:10.3390/ijms26115037)
Supplement: Supplementary file 1 [file ijms-26-05037-s001.zip › ijms-3563918-supplementary.pdf]

Supplementarry Table S1. List of the variants of CLN2 gene detected using ONT sequencing platform.

Case	Variant Location	RS No:	Var. Type	Var. Classification	Status	HGVSc	HGVSp	Zygocity
1.1	11_6612918_C/T	rs7488	SNV	3' UTR variant	B	c.*1628G>A		hom
Index	11_6612958_G/A	rs7487	SNV	3' UTR variant	B	c.*1588C>T		hom
	11_6616287_A/G	rs7943955	SNV	intron variant	B	c.1075+28T>C		hom
	11_6616690_T/C	rs119455958	SNV	missense variant	LP	c.857A>G	p.Ala513GlnfsTer6, ENSP00000437066.1	hom
	11_6618120_T/C	rs2734718	SNV	intron variant	B	c.230-344A>G		hom
Index	11_6613428_C/G	rs1045450	SNV	3' UTR variant	B	c.*1118G>C		hom
	11_6614875_T/A	rs1128396	SNV	synonymous variant	B	c.1542A>T	p.Gly514=, ENSP00000437066.1	hom
	11_6615504_C/A		SNV	stop don	LP	c.1204G>T	p.Glu402Ter, ENSP00000398136.3	hom
	11_6616273_G/A	rs2072651	SNV	intron variant	B	c.1075+42C>T		hom
	11_6616287_A/G	rs7943955	SNV	intron variant	B	c.1075+28T>C		hom
2.2	11_6612918_C/T	rs7488	SNV	3' UTR variant	B	c.*1628G>A		het
Mother	11_6613428_C/G	rs1045450	SNV	3' UTR variant	B	c.*1118G>C		het
	11_6614875_T/A	rs1128396	SNV	synonymous variant	B	c.1542A>T	p.Gly514=, ENSP00000437066.1	???
	11_6615504_C/A		SNV	stop codon	LP	c.1204G>T	p.Glu402Ter, ENSP00000398136.3	het
	11_6616273_G/A	rs2072651	SNV	intron variant	B	c.1075+42C>T		het
	11_6616287_A/G	rs7943955	SNV	intron variant	B	c.1075+28T>C		hom
2.3	11_6612918_C/T	rs7488	SNV	3' UTR variant	B	c.*1628G>A		hom
Sibling	11_6613428_C/G	rs1045450	SNV	3' UTR variant	B	c.*1118G>C		hom
	11_6614875_T/A	rs1128396	SNV	synonymous variant	B	c.1542A>T	p.Gly514=, ENSP00000437066.1	hom
	11_6615504_C/A		SNV	stop codon	LP	c.1204G>T	p.Glu402Ter, ENSP00000398136.3	het
	11_6616273_G/A	rs2072651	SNV	intron variant	B	c.1075+42C>T		hom
	11_6616287_A/G	rs7943955	SNV	intron variant	B	c.1075+28T>C		hom
2.4	11_6612918_C/T	rs7488	SNV	3' UTR variant	B	c.*1628G>A		hom
Sibling	11_6613428_C/G	rs1045450	SNV	3' UTR variant	B	c.*1118G>C		hom
	11_6614875_T/A	rs1128396	SNV	synonymous variant	B	c.1542A>T	p.Gly514=, ENSP00000437066.1	??
	11_6615504_C/A		SNV	stop codon	LP	c.1204G>T	p.Glu402Ter, ENSP00000398136.3	het
	11_6616273_G/A	rs2072651	SNV	intron variant	B	c.1075+42C>T		hom
	11_6616287_A/G	rs7943955	SNV	intron variant	B	c.1075+28T>C		hom
3.1	11_6617040_G/A	rs119455955	SNV	stop codon	P	c.622C>T	p.Arg208Ter, ENSP00000493574.1	hom
Index	11_6618120_T/C	rs2734718	SNV	intron variant	B	c.230-344A>G		hom
	11_6616287_A/G	rs7943955	SNV	intron variant	B	c.1075+28T>C		hom
	11_6612918_C/T	rs7488	SNV	3' UTR variant	B	c.*1628G>A		hom
	11_6612958_G/A	rs7487	SNV	3' UTR variant	B	c.*1588C>T		hom
	11_6617275_A/T	rs1800738	SNV	intron variant	B	c.508+26T>A		hom
4.1	11_6612958_G/A	rs7487	SNV	3' UTR variant	B	c.*1588C>T		het
Index	11_6613428_C/G	rs1045450	SNV	3' UTR variant	B	c.*1118G>C		het
	11_6614875_T/A	rs1128396	SNV	synonymous variant	B	c.1542A>T	p.Gly514=, ENSP00000437066.1	het
	11_6616273_G/A	rs2072651	SNV	intron variant	B	c.1075+42C>T		het
	11_6616287_A/G	rs7943955	SNV	intron variant	B	c.1075+28T>C		hom

	11_6617040_G/A	rs119455955	SNV	stop codon	P	c.622C>T	p.Arg208Ter, ENSP00000493574.1	het
	11_6618780_T/C	rs368709098	SNV	Aberrant splicing , 3rd exon skipped	P	c.225A>G	p.Gln75 =	het
	11_6618120_T/C	rs2734718	SNV	intron variant	B	c.230-344A>G		hom
4.2	11_6612958_G/A	rs7487	SNV	3' UTR variant	B	c.*1588C>T		het
Mother	11_6613428_C/G	rs1045450	SNV	3 ' UTR variant	B	c.*1118G>C		het
	11_6616287_A/G	rs7943955	SNV	intron variant	B	c.1075+28T>C		hom
	11_6616273_G/A	rs2072651	SNV	intron variant	B	c.1075+42C>T		het
	11_6617275_A/T	rs1800738	SNV	intron variant	B	c.508+26T>A		het
	11_6618780_T/C	rs368709098	SNV	Aberrant splicing , 3rd ex	P	c.225A>G	p.Gln75 =	het
4.3	11_6612958_G/A	rs7487	SNV	3' UTR variant	B	c.*1588C>T		het
Father	11_6613428_C/G	rs1045450	SNV	3' UTR variant	B	c.*1118G>C		het
	11_6616273_G/A	rs2072651	SNV	intron variant	B	c.1075+42C>T		het
	11_6616287_A/G	rs7943955	SNV	intron variant	B	c.1075+28T>C		hom
	11_6617040_G/A	rs119455955	SNV	stop codon	P	c.622C>T	p.Arg208Ter, ENSP00000493574.1	het
5.1	11_6612918_C/T	rs7488	SNV	3' UTR variant	B	c.*1628G>A		hom
Index	11_6612958_G/A	rs7487	SNV	3' UTR variant	B	c.*1588C>T		hom
	11_6616287_A/G	rs7943955	SNV	intron variant	B	c.1075+28T>C		hom
	11_6617040_G/A	rs119455955	SNV	stop codon	P	c.622C>T	p.Arg208Ter, ENSP00000493574.1	hom
6.1	11_6612918_C/T	rs7488	SNV	3' UTR variant	B	c.*1628G>A		hom
Index	11_6613428_C/G	rs1045450	SNV	3' UTR variant	B	c.*1118G>C		hom
	11_6615504_C/A		SNV	stop codon	LP	c.1204G>T	p.Glu402Ter, ENSP00000398136.3	hom
	11_6616273_G/A	rs2072651	SNV	intron variant	B	c.1075+42C>T		hom
	11_6618120_T/C	rs2734718	SNV	intron variant	B	c.230-344A>G		hom
6.2	11_6612918_C/T	rs7488	SNV	3' UTR variant	B	c.*1628G>A		hom
Mother	11_6613428_C/G	rs1045450	SNV	3' UTR variant	B	c.*1118G>C		het
	11_6615504_C/A		SNV	stop codon	LP	c.1204G>T	p.Glu402Ter, ENSP00000398136.3	het
	11_6616273_G/A	rs2072651	SNV	intron variant	B	c.1075+42C>T		het
	11_6618120_T/C	rs2734718	SNV	intron variant	B	c.230-344A>G		hom
	11_6616287_A/G	rs7943955	SNV	intron variant	B	c.1075+28T>C		hom
	11_6618827_T/-		deletion	frameshift variant	VUS	c.178del	p.Arg60AspfsTer21, ENSP00000493706.1	het
7.1	11_6615669_G/C	rs1800745	SNV	intron variant	B	c.1146-107C>G		hom
Index	11_6615069_A/G	rs1800723	SNV	intron variant	B	c.1426-78T>C		hom
	11_6616287_A/G	rs7943955	SNV	intron variant	B	c.1075+28T>C		hom
	11_6617040_G/A	rs119455955	SNV	stop codon	P	c.622C>T	p.Arg208Ter, ENSP00000493574.1	hom
	11_6618120_T/C	rs2734718	SNV	intron variant	B	c.230-344A>G		hom
	11_6619206_G/C	rs775155638	SNV	missense variant	VUS	c.79C>G		het
7.2	11_6615669_G/C	rs1800745	SNV	intron variant	B	c.1146-107C>G		het
Father	11_6615069_A/G	rs1800723	SNV	intron variant	B	c.1426-78T>C		het
	11_6616287_A/G	rs7943955	SNV	intron variant	B	c.1075+28T>C		hom
	11_6617040_G/A	rs119455955	SNV	stop codon	P	c.622C>T	p.Arg208Ter, ENSP00000493574.1	het
	11_6618120_T/C	rs2734718	SNV	intron variant	B	c.230-344A>G		hom
	11_6619206_G/C	rs775155638	SNV	missense variant	VUS	c.79C>G		het
7.3	11_6612958_G/A	rs7487	SNV	3' UTR variant	B	c.*1588C>T		het
Mother	11_6615069_A/G	rs1800723	SNV	intron variant	B	c.1426-78T>C		het

	11_6615669_G/C	rs1800745	SNV	intron variant	B	c.1146-107C>G		het
	11_6616287_A/G	rs7943955	SNV	intron variant	B	c.1075+28T>C		hom
	11_6617040_G/A	rs119455955	SNV	stop codon	P	c.622C>T	p.Arg208Ter, ENSP00000493574.1	het
	11_6618120_T/C	rs2734718	SNV	intron variant	B	c.230-344A>G		hom
7.4	11_6618120_T/C	rs2734718	SNV	intron variant	B	c.230-344A>G		hom
Sibling	11_6616287_A/G	rs7943955	SNV	intron variant	B	c.1075+28T>C		hom
	11_6616273_G/A	rs2072651	SNV	intron variant	B	c.1075+42C>T		het
7.5	11_6615669_G/C	rs1800745	SNV	intron variant	B	c.1146-107C>G		het
Sibling	11_6616287_A/G	rs7943955	SNV	intron variant	B	c.1075+28T>C		hom
	11_6617040_G/A	rs119455955	SNV	stop codon	P	c.622C>T	p.Arg208Ter, ENSP00000493574.1	het
	11_6618120_T/C	rs2734718	SNV	intron variant	B	c.230-344A>G		hom
	11_6619206_G/C	rs775155638	SNV	missense variant	VUS	c.79C>G		het
7.6	11_6616287_A/G	rs7943955	SNV	intron variant	B	c.1075+28T>C		hom
Uncle	11_6613428_C/G	rs1045450	SNV	3' UTR variant	B	c.*1118G>C		het
	11_6612958_G/A	rs7487	SNV	3' UTR variant	B	c.*1588C>T		het
7.7	11_6615669_G/C	rs1800745	SNV	intron variant	B	c.1146-107C>G		het
Uncle	11_6615069_A/G	rs1800723	SNV	intron variant	B	c.1426-78T>C		het
	11_6616273_G/A	rs2072651	SNV	intron variant	B	c.1075+42C>T		het
	11_6617040_G/A	rs119455955	SNV	stop codon	P	c.622C>T	p.Arg208Ter, ENSP00000493574.1	het
7.9	11_6612958_G/A	rs7487	SNV	3' UTR variant	B	c.*1588C>T		het
Uncle’s	11_6612918_C/T	rs7488	SNV	3' UTR variant	B	c.*1628G>A		het
Wife	11_6615669_G/C	rs1800745	SNV	intron variant	B	c.1146-107C>G		het
	11_6616287_A/G	rs7943955	SNV	intron variant	B	c.1075+28T>C		hom
	11_6618120_T/C	rs2734718	SNV	intron variant	B	c.230-344A>G		hom
7.1	11_6612918_C/T	rs7488	SNV	3' UTR variant	B	c.*1628G>A		hom
Cousin	11_6612958_G/A	rs7487	SNV	3' UTR variant	B	c.*1588C>T		het
	11_6616287_A/G	rs7943955	SNV	intron variant	B	c.1075+28T>C		hom
	11_6618120_T/C	rs2734718	SNV	intron variant	B	c.230-344A>G		hom
7.11	11_6618120_T/C	rs2734718	SNV	intron variant	B	c.230-344A>G		hom
Cousin	11_6612918_C/T	rs7488	SNV	3' UTR variant	B	c.*1628G>A		hom
	11_6616287_A/G	rs7943955	SNV	intron variant	B	c.1075+28T>C		hom

A

c.857A>G

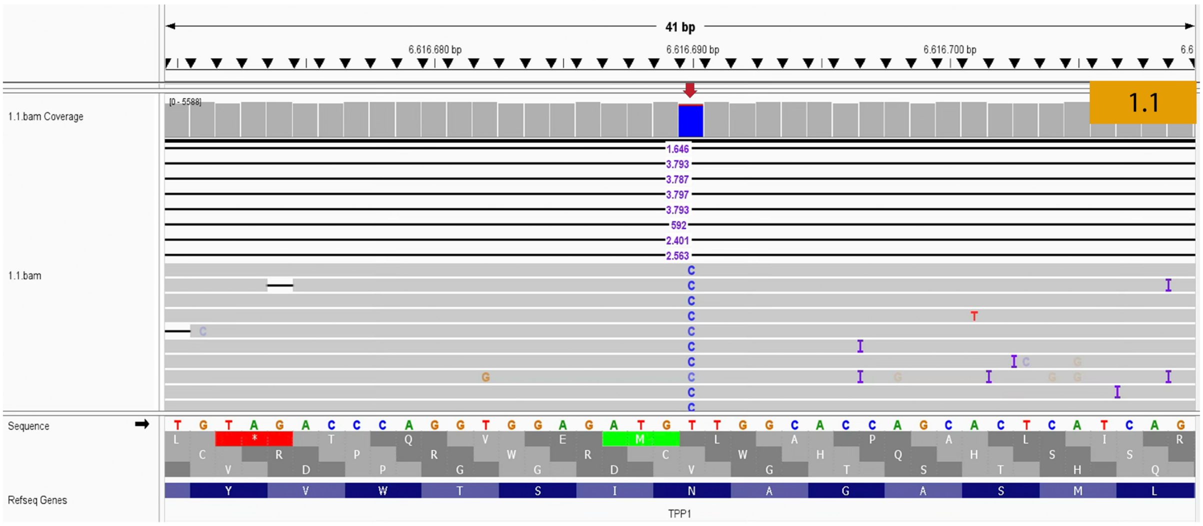

41 bp
6.616.680 bp
6.616.690 bp
6.616.700 bp
[0 - 5588]
1.1
1.1.bam Coverage
1.646
3.793
3.787
3.797
3.793
592
2.401
2.563
1.1.bam
Sequence
Refseq Genes
TPP1

B

c.225A>G

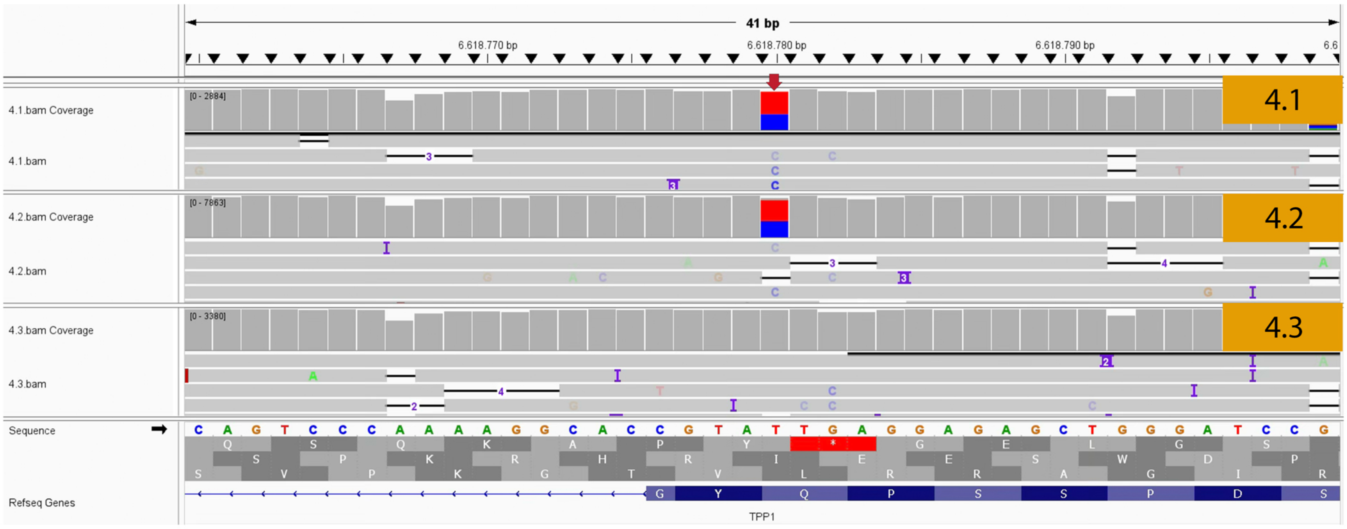
41 bp
6.618.770 bp
6.618.780 bp
6.618.790 bp
4.1.bam Coverage
[0 - 2884]
4.1
4.1.bam
4.2.bam Coverage
[0 - 7863]
4.2
4.2.bam
4.3.bam Coverage
[0 - 3380]
4.3
4.3.bam
Sequence
C A G T C C C C A A A A G G C A C C G T A T T G A G G A G A G C T G G G A T C C G
Refseq Genes
TPP1

C

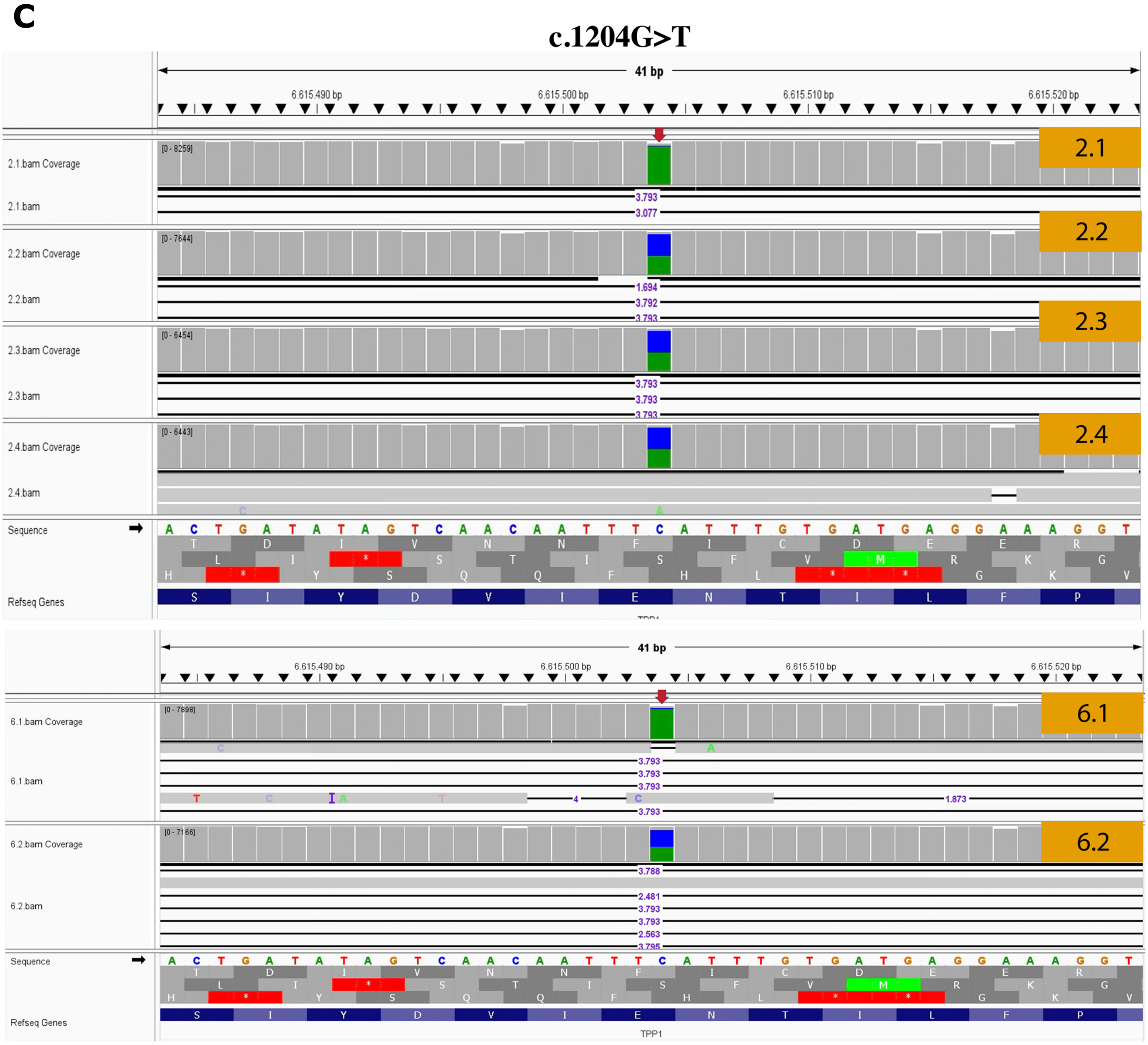
c.1204G>T
41 bp
6.615.490 bp
6.615.500 bp
6.615.510 bp
6.615.520 bp
2.1
2.1.bam Coverage
2.1.bam
2.2
2.2.bam Coverage
2.2.bam
2.3
2.3.bam Coverage
2.3.bam
2.4
2.4.bam Coverage
2.4.bam
Sequence
Refseq Genes
6.1
6.1.bam Coverage
6.1.bam
6.2
6.2.bam Coverage
6.2.bam
TPP1

D

c.622C>T

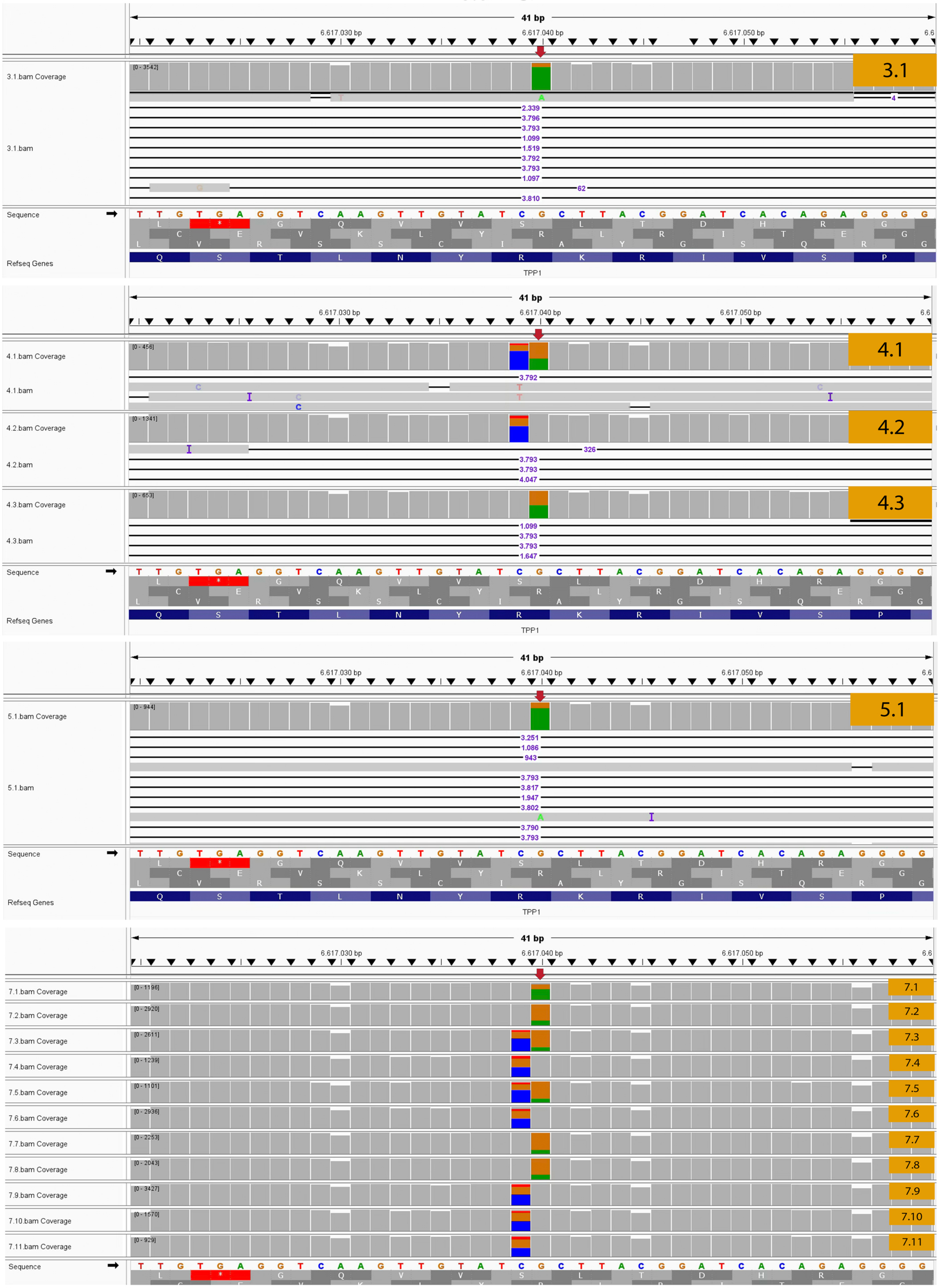
41 bp
6.617.030 bp
6.617.040 bp
6.617.050 bp
3.1.bam Coverage
3.1.bam
3.1
4.1.bam Coverage
4.1.bam
4.1
4.2.bam Coverage
4.2.bam
4.2
4.3.bam Coverage
4.3.bam
4.3
5.1.bam Coverage
5.1.bam
5.1
7.1.bam Coverage
7.2.bam Coverage
7.3.bam Coverage
7.4.bam Coverage
7.5.bam Coverage
7.6.bam Coverage
7.7.bam Coverage
7.8.bam Coverage
7.9.bam Coverage
7.10.bam Coverage
7.11.bam Coverage
7.1
7.2
7.3
7.4
7.5
7.6
7.7
7.8
7.9
7.10
7.11
Sequence
Refseq Genes
TPP1

Supplenmentary Figure S1. Integrative Genomics Viewer (IGV) illustrations showing the (**A**) c857A>G, (**B**) c.225A>G, (C) c.1204G>T, (D) c.622C>T variants.

(**A**) The variant c857A>G is homozygous in the individual 1.1 (index patient). (**B**) The c.225A>G variant is detected in the index patient (4.1) and the mother (4.2), while the father (4.3) is non-carrier. (**C**) IGV tracts of the variant c.1204G>T in family 2 and 6. In family 2, the affected individual 2.1 is homozygous for the variant, while the mother (2.2) and siblings (2.3 and 2.4) are heterozygous. In family 6, the index patient (6.1) is homozygous and the mother (6.2) is heterozygous. (**D**) The c.622C>T variant is detected in families 3, 5 and 7. Index patients 3.1, 5.1 and 7.1 are homozygous for the variant, while the index patient (4.1) and the father (4.3) are heterozygous. In family 7, carriers include the father (7.2), mother (7.3), a brother (7.5), the maternal aunt (7.7) and the maternal uncle (7.8).
